# The brain-gut-microbiota axis in the treatment of neurologic and psychiatric disorders

**DOI:** 10.1055/s-0043-1767818

**Published:** 2023-07-04

**Authors:** Maria Fernanda Naufel, Giselle de Martin Truzzi, Caroline Marcantonio Ferreira, Fernando Morgadinho Santos Coelho

**Affiliations:** 1 Universidade Federal de São Paulo, Departamento de Fisiologia, São Paulo SP, Brazil.; 2 Universidade Federal de São Paulo, Departamento de Psicobiologia, São Paulo SP, Brazil.; 3 Universidade Federal de São Paulo, Departamento de Ciências Farmacêuticas, São Paulo SP, Brazil.; 4 Universidade Federal de São Paulo, Departamento de Neurologia e Neurocirurgia, São Paulo SP, Brazil.

**Keywords:** Gastrointestinal Microbiome, Dysbiosis, Central Nervous System, Therapeutics, Microbioma Gastrointestinal, Disbiose, Sistema Nervoso Central, Terapêutica

## Abstract

The human gut microbiota is a complex ecosystem made of trillions of microorganisms. The composition can be affected by diet, metabolism, age, geography, stress, seasons, temperature, sleep, and medications. The increasing evidence about the existence of a close and bi-directional correlation between the gut microbiota and the brain indicates that intestinal imbalance may play a vital role in the development, function, and disorders of the central nervous system. The mechanisms of interaction between the gut-microbiota on neuronal activity are widely discussed. Several potential pathways are involved with the brain-gut-microbiota axis, including the vagus nerve, endocrine, immune, and biochemical pathways. Gut dysbiosis has been linked to neurological disorders in different ways that involve activation of the hypothalamic-pituitary-adrenal axis, imbalance in neurotransmitter release, systemic inflammation, and increase in the permeability of the intestinal and the blood-brain barrier. Mental and neurological diseases have become more prevalent during the coronavirus disease 2019pandemic and are an essential issue in public health globally. Understanding the importance of diagnosing, preventing, and treating dysbiosis is critical because gut microbial imbalance is a significant risk factor for these disorders. This review summarizes evidence demonstrating the influence of gut dysbiosis on mental and neurological disorders.

## Resumo

A microbiota intestinal humana é um ecossistema complexo feito de trilhões de microrganismos, cuja composição pode ser afetada pela dieta, pelo metabolismo, pela idade, geografia, pelo estresse, pelas estações do ano, pela temperatura, pelo sono e por medicamentos. A crescente evidência sobre a existência de uma correlação estreita e bidirecional entre a microbiota intestinal e o cérebro indica que o desequilíbrio intestinal pode desempenhar um papel vital no desenvolvimento, na função e nos distúrbios do sistema nervoso central. Os mecanismos de interação entre a microbiota intestinal e a atividade neuronal são amplamente discutidos. Várias vias potenciais estão envolvidas com o eixo microbiota-intestino-cérebro, incluindo o nervo vago e as vias endócrinas, imunes e bioquímicas. A disbiose intestinal tem sido associada a distúrbios neurológicos de diferentes maneiras que envolvem a ativação do eixo hipotálamo-hipófise-adrenal, o desequilíbrio na liberação de neurotransmissores, a inflamação sistêmica e o aumento da permeabilidade das barreiras intestinal e hematoencefálica. As doenças mentais e neurológicas tornaram-se mais prevalentes durante a pandemia de *coronavirus disease 2019* e são uma questão global essencial na saúde pública. Compreender a importância de diagnosticar, prevenir e tratar a disbiose é fundamental porque o desequilíbrio microbiano intestinal é um fator de risco significativo para esses distúrbios. Esta revisão resume as evidências que demonstram a influência da disbiose intestinal em distúrbios mentais e neurológicos.

## INTRODUCTION

There are 100 billion neurons in the human brain and 500 million in our gut. The gut is connected to our central nervous system (CNS) and influences brain function through nerves, blood circulation, and lymphatic ways. Another essential component of the interaction between gut and brain is the gut microbiota. This microbiota is a complex ecosystem configured by trillions of microorganisms. The gut microbiota is affected by diet, metabolism, age, geography, stress, seasons, temperature, sleep, medications, and others. A correct balance in the composition of the gut microbiota is vital for mental and physical health, and for the prevention and management of several diseases. The interaction between these numerous structures in the brain and gastrointestinal tract involving the microbiota encompasses the concept of the brain-gut microbiota axis (BGMA), a target of increasing clinical interest. The relationship between the higher prevalence of neurological, psychiatric, and sleep disorders and changes in gut microbiota is being explored to unravel the mechanisms of action of this relationship so that treatment strategies can be developed. 1

In 2007, the Human Microbiome Project began to unravel the abundance, diversity, and function of genes present in the microbiota and their interference in health and disease. 2 Approximately 15 years of studies have passed, and the knowledge acquired in this area is still insufficient to treat neurological and mental disorders effectively. We know that lifestyle habits such as increased consumption of processed foods and decreased consumption of complex carbohydrates, fruits and vegetables alter the composition of the intestinal microbiota, leading to a condition called dysbiosis. Gut dysbiosis is an imbalance of the intestinal microbiome, which involves the loss of commensal species and increase in the pathogenic microorganism. This microbiome disharmony can alter nutrient availability, trigger chronic inflammation, weaken the human immune system, and begin with many types of diseases. Mental and neurological diseases have become more prevalent during the coronavirus disease 2019 (COVID-19) pandemic and are an essential issue in public health globally. Considering that dysbiosis is a significant risk factor for these disorders, understanding the importance of diagnosing, preventing, and treating dysbiosis is critical. This review summarizes evidence demonstrating the influence of gut dysbiosis on mental and neurological pathophysiology. We also explore dysbiosis diagnosis and the potential of the gut microbiota as a therapeutic target for brain disorders. 3

## METHODS

A literature review was performed to identify relevant studies on the role of the microbiome in neurological disorders, including brain, psychiatric, and sleep disorders, by searching the electronic databases of PubMed, ScienceDirect, and Scopus.

To provide a broader perspective on this issue, we reviewed the literature published between 2000 and 2022 in English. Two authors (M. F. N. and F. M. S. C.) conducted the study selection process independently. They identified articles by title, abstract, and full text. Any disagreements were discussed and resolved.

### Pathways involved with brain-gut microbiota axis

The discussion about putative mechanisms of the indirect and direct impact of the microbiota on neuronal activity is widespread. There are several potential pathways involved with BGMA. First, the neural way is the fastest communication channel between the brain, gut, and microbiota. Moreover, microbiota can affect the CNS's function bidirectionally via the vagus nerve. An article published in 2011 showed that the vagus nerve plays a significant role in the gastrointestinal tract and CNS. The mechanism involved depends on alterations in brain gamma-aminobutyric acid (GABA)mRNA. Also, the neurochemical and behavioral effects of nonpathogenic bacteria treatment depend on the vagus, considering vagotomized mice do not show alterations. 4 In addition, human studies involving nonpathogenic bacteria administration confirmed the potential translatability of such findings. How gut bacteria can use the vagus nerve to communicate with the CNS remains to be clarified. 5

Secondly, the endocrine pathway (e.g., circulation of neuropeptides or hormones) interacts with these distant structures gut and brain. Any neuroactive substances (precursors and neurotransmitters) in the gut can affect the neuronal activity in the brain. Many neurotransmitter precursors from the gut can cross through the blood-brain barrier (BBB), influencing various neurological electrophysical and biochemical interactions. Third, the immune pathway is influenced by the gut microbiota as components of the microbiota interact with cells and their receptors in the gut mucosa all the time, stimulating homeostatic control of immune responses, which are important for the symbiotic relationship between the microbiota and the host. However, some inflammatory triggers, such as gut pathogenic bacteria or exposure to a toxic substance, can activate immune cells, which may modulate neuronal activity via the release of neurotransmitters and cytokines. 6

Finally, various microbiota bacteria can metabolize tryptophan, limiting its use as a precursor of indole, melatonin, and serotonin. Low levels of serotonin have been linked to mental and neurological disorders. Besides these biochemical pathways, gut microbiota can produce metabolites such as short-chain fatty acid (SCFA) and synthesize other neurotransmitters. Short-chain fatty acid can cross the BBB contributing to the GABA metabolic cycle in the hypothalamus. Interestingly, 11C- and 13C-labeled acetate from the gut acts in the hypothalamus, stimulating tricarboxylic acid, glutamate-glutamine, and GABA transcellular cycles. Also, microbiota produces enzymes that can facilitate the synthesis of neurotransmitters or their precursors, such as glutamate, GABA, dopamine, and serotonin. 7

**Figure 1:**
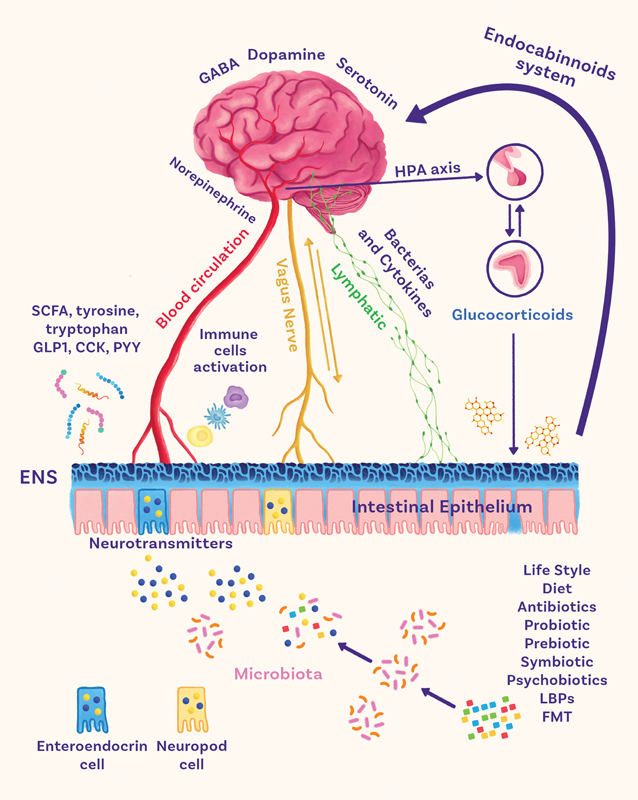
The brain-gut-microbiota axis. Abbreviations: CCK, cholecystokinin; ENS, enteric nervous system; FMT, fecal microbiota transplantation; GLP1, glucagon-like peptide 1; HPA, hypothalamic–pituitary–adrenal; LBPs, live biotherapeutic products; PYY, peptide YY; SCFA, short-chain fatty acids.

Usually, these neurotransmitters do not penetrate the BBB, but capillary endothelial cells can actively transport many neurotransmitter precursors into the brain. The concentration of many of them, such as tyrosine and tryptophan, contribute to neurotransmitter-producing. Dysbiosis can disrupt these pathways inducing mental and neurological diseases ( Figure 1 ). 7

#### Gut dysbiosis diagnosis

Feces samples are usually collected for the human gut microbiome analysis. However, fecal samples are just a proxy of the human intestinal microbiota, and researchers comprehend that might be an essential distinction between the microbial composition of feces and intestinal mucosa. Another way to assess gut microbiota is by endoscopic biopsy, which can investigate distinct areas of the gastrointestinal (GI) tract. However, biopsies are invasive methods to determine the microbiome; thus, it is not appropriate to investigate healthy individuals. 8

Other possible sampling methods for collecting gut microbiota are luminal brush, laser capture microdissection, catheter aspiration, intelligent capsule, surgery, ileostomy, and fluorescence in situ hybridization (FISH). Nevertheless, they are invasive, inappropriate for healthy individuals, and some are unsuitable for most studies and mainly used for commercial analysis. 8

After sample collection, it is necessary to apply an established methodology to extract the microbial DNA, to assess and quantify the gut microbiota composition. There are some offered methods to determine and quantify dysbiosis. A recent study has classified the dysbiosis indexes into five categories according to the methodology: Large-scale bacterial markers; relevant taxon-based plans; neighborhood classification; Random Forest prediction, and combined α and β diversity. 9

Although diagnosing gut dysbiosis is essential to elucidate and treat numerous diseases, including neurological disorders (such as Alzheimer, Parkinson, multiple sclerosis, mood disorders, and others), for many reasons, it is still a challenge for physicians to assess intestinal microbiota imbalance in their clinical practices. Faulty sampling methods are part of the adversity for converting scientific studies into medical interventions. Moreover, the microbiome is highly complex, resulting in datasets that demand refined statistical analysis, which cannot afford commercial purposes. Besides, the diagnosis methods currently available are costly. 8

Thus, prospective studies are still necessary to improve from a descriptive phase of research, to better elucidate and uncomplicate dysbiosis assessment and, other than that, conclude about types of dysbiosis (e.g., loss of beneficial bacteria, overgrowth of pathogenic bacteria, reduction of bacterial diversity) and if each type has equally or distinct weights to trigger or aggravate different categories of diseases. 10

### Neurological disorders

#### Alzheimer’s disease

Alzheimer’s disease (AD) is a degenerative and progressive cognitive impairment disease, with no treatment available to interrupt the degenerative process and revive degenerated brain cells, and it is associated with the malfunction of several neurotransmitters, such as acetylcholine, dopamine, GABA, serotonin, glutamate, and norepinephrine. The concentration of these neurotransmitters depends on bacterial taxa such as *E. coli* , *Bacteroides* , *Eubacterium* , and *Bifidobacterium* . 11

Studies have reported dysbiosis in patients with AD. There is an imbalance in gut microbiota (rises of *Bacteroides* , *Escherichia/Shigella* , and *Ruminococcus* and reduction of *Dialister* and *E. rectale* , *Bifidobacterium* ) influencing the progress of AD due to dysregulation of the synthesis of neurotransmitters and the precursors of them. 12 A recent study characterized an abundance of several bacteria taxa in mild AD patients ( *Anaerostipes* , *Mitsuokella* , *Prevotella, Bosea* , *Fusobacterium* , *Anaerotruncus* , *Clostridium* , and *Coprobacillus* ), and there was an abundance of an emerging probiotic ( *Akkermansia* ) and a traditional probiotic ( *Bifidobacteria* ). The authors defend this pattern of the fecal microbiome in mild AD patients. 13

The treatment of AD patients is related to a pharmacological treatment with acetylcholinesterase inhibitors (tacrine, donepezil, rivastigmine, and galantamine), N-methyl-D-aspartate (NMDA) receptor antagonist (memantine), and the recent aducanumab. 14

However, there is a growing interest in dietary interventions to treat AD patients with fermented foods and drug development. The diagnosis of this gut dysbiosis is not a reality in real life yet. However, potential fermented food products have been studied with safe long-term eating to control the disease outcome and minimize commercial drug side effects and toxicity. Recently, a systematic review on the impact of probiotics in animal AD studies demonstrated that although the effect of probiotic administration on Aβ remains ambiguous, probiotics such as *B. longum* (NK46), *C. butyricum* , and the mixture SLAB51 have exhibited promising effects to relieve AD symptoms 15

#### Parkinson’s disease

Parkinson’s disease (PD) is a neurodegenerative disorder with dysregulation of the dopamine system due to aggregation of insoluble α-synuclein (µ-syn) protein in vulnerable populations of neurons and glia. Gut dysbiosis has also been observed in PD patients (increase of *Akkermansia, Catabacter* , *Lactobacillus* , *Bifidobacterium, Bifidobacteriaceae* , *Ruminococcaceae* , *Verrucomicrobiaceae* , *Christensenellaceae* , and decrease of *Roseburia* , *Faecalibacterium* , *Lachnospiraceae ND3007* group, *Prevotellaceae* , *Blautia* , *Coprococcus* , and *Lachnospira* ). 13

The other factors associated with gut microbiota were constipation severity, physical activity, and pharmacological therapies associated with β diversity. They concluded that the gut microbiota combined with macronutrient intake could predict the development of PD. Recent reports on the gut microbiome in PD showed no differences in the fungi abundance, decreased total virus richness, and significantly increased *Lactobacillaceae* abundance, consistent with the overrepresentation of *Lactobacillales* . 16

The pathological process suggested for PD is active retrograde transport of ∝-syn from the enteric nervous system (ENS) to the CNS by the dorsal motor nucleus of the vagus nerve (DMNV). An extracellular amyloid protein named “curli” (secreted by *Escherichia coli* ) can be implicated, inducing neuronal deposition of a-syn in the gut and promoting neuroinflammation. [80] Experimental animal models with gut dysbiosis show that gut microbiota is related to motor impairments and gastrointestinal (GI) dysfunction. The microbial molecules, via the gut–brain, impact microglia activation, neuroinflammation, and ∝-syn aggregation. 17

*Enterococcus* and *Bacillus* are bacteria capable of synthesizing dopamine in gut microbiota. The intraduodenal infusion of levodopa-carbidopa intestinal gel (LCIG) is related to an increased relative abundance of the *Enterobacteriaceae* family, as well as to lower absorption of L-dopa in PD patients infected by *Helicobacter pylori* . Indeed, there is a bidirectional approach between medications and gut microbiota in PD patients. 18

Authors have demonstrated the influence of *E. faecalis* in the activity of levodopa decarboxylation with potential clinical response influence. However, a recent study showed that Levodopa was not associated with changes in microbiota composition and concluded that more extensive and longer-term studies must be done. 19

Several clinical trials with fecal microbiota transplantation from healthy controls to patients with PD have demonstrated significant effects on attenuating the motor symptoms, meanwhile improving non-motor symptoms such as the quality of sleep and life and relieving anxiety and depression constipation symptoms in PD. 20

#### Multiple sclerosis

Multiple sclerosis (MS) is a prevalent neurological disease, which is more likely to occur in Europe, North America, and Australasia. The risk factors involved in the pathology are highly complex and combine genetic variants and environmental factors. Evidence supports that the gut microbiota composition of patients with MS suffers alteration, as several studies observed that the intestinal microbiome profile of these patients differs from healthy controls. 21

The modification of the bacterial population detected in the gut of patients with MS seems to lead to a proinflammatory state. At the same time, dysbiosis increases the BBB permeability. It results in CNS inflammation and neurodegeneration, which might culminate in the development of MS. 22

An overview of clinical trials that evaluated the gut microbiota composition of patients with MS observed that, compared with healthy controls, these patients had increased concentrations of *Pedobacteria* , *Flavobacterium* , *Pseudomonas* , *Mycoplana* , *Acinetobacter* , and *Eggerthella* , *Dorea* , *Blautia* , *Streptococcus* , and *Akkermansia* . In contrast, their gut microbiota presents lower amounts of *Prevotella, Bacteroides* , *Parabacteroides* , *Haemophilus* , *Sutterela* , *Adlercreutzia* , *Croprobacillus* , *Lactobacillus* , *Clostridium* , *Anaerostipes* , and *Faecalibacterium* , showing that dysbiosis is a fact in patients with MS. The authors suggest that the dysbiosis treatment seems to reduce inflammation and reactivate the immune system by regulating the T lymphocytes effect in humans. 23

#### Epilepsy

The gut microbiota of patients with epilepsy has also been studied. As shown in other neurological disorders, it seems to be altered compared with healthy controls, which increases inflammation and may influence drug-resistant seizures. 24

In a recent longitudinal study in drug-naïve children with epilepsy, researchers observed that the intestinal microbiome of these patients had an increased abundance of *Akkermansia ssp.* and *Proteobacteria* and a decreased number of *Faecalibacterium ssp* , suggesting that dysbiosis and taxonomy may happen in the gut of patients with epilepsy. However, clinical trials evaluating the gut microbiota of patients with epilepsy are still scarce, and further studies must be performed to confirm these findings ( Table 1 ). 25

**Table 1:** Summary of studies assessing the response to probiotic administration for treating brain and psychologic disorders

Neurological disorder	Subjects	Probiotic treatment formula	Treatment duration	Treatment effects	Level of evidence for dysbiosis treatment*	References
**Alzheimer’s disease (AD)**	79 patients with AD, age range between 55–100 years. Patients were randomly assigned to receive either probiotic plus selenium (200 μg/day), only selenium (200 μg/day) or placebo.	*Lactobacillus (L.) acidophilus, Bifidobacterium (B.) bifidum,* and *B. longum*	12 weeks	Probiotic with selenium co-supplementation improved cognitive function and regulates metabolic abnormality and oxidative stress.	Level II	Tamtaji et al. (2019) 79
	48 people with AD (age range between 65–90 years old) were divided into control group ( *n* = 23) and probiotic group ( *n* = 25).	*L. fermentum* , *L. plantarum* , *B. lactis* , *L. acidophilus* , *B. bifidum* , and *B. longum*	12 weeks	Observed regulation of serum metabolites, however, authors concluded that cognitive and biochemical parameters are insensitive to the probiotic supplementation. Moreover, probiotic formulation and dosage, the severity of AD and time of administration deeply affects results treatment.	Level III	Agahi et al. (2018) 76
**Parkinson’s disease (PD)**	120 patients with PD were randomly assigned (2:1) to either a fermented milk containing multiple probiotic strains plus prebiotic fiber ( *n* = 80, aged 71.8 ± 7.7), or placebo ( *n* = 40, aged 69.5 ± 10.3).	*Streptococcus (S.) salivarius subsp thermophilus* , *Enterococcus (E.) faecium* , *L. rhamnosus GG* , *L. acidophilus, L. plantarum* , *L. paracasei* , *L. delbrueckii subsp bulgaricus* , and *B. (breve* and *animalis subsp lactis).* **Prebiotic treatment:** *Fructooligosaccharides*	4 weeks	In the treatment group, there was a significant increase in the number of complete bowel movements, improvements in bowel frequency, stool consistency, and frequency of laxative usage. Therefore, probiotic supplementation improved constipation in PD patients.	Level II	Barichella et al. (2016) 55
**Multiple sclerosis (MS)**	65 patients with MS (18–50 years old) were randomized into intervention ( *n* = 32) and received 2 multi-strain probiotic capsules daily, or into control group ( *n* = 33).	*Bacillus subtilis PXN 21* , *B. bifidum PXN 23* , *B. breve PXN 25* , *B. infantis PXN 27* , *B. longum PXN 30* , *L. acidophilus PXN 35* , *L. delbrueckii ssp. bulgaricus PXN 39* , *L. casei PXN 37* , *L. plantarum PXN 47* , *L. rhamnosus PXN 54* , *L. helveticus PXN 45* , *L. salivarius PXN 57* , *L. lactis ssp. lactis PXN 63* , *S. thermophilus PXN 66,* plus cellulose and vegetable capsule ( *Hydroxypropylmethyl cellulose* ).	6 months	Probiotic supplementation for six months resulted in greater improvement in mental health parameters of patients with MS, significantly reducing depression severity, improving depression symptoms and life quality, reducing fatigue, and improving inflammatory biomarkers.	Level II	Rahimlou et al. (2022) 78
	60 MS patients (18–55 years old) were randomly allocated into probiotic ( *n* = 30) or placebo ( *n* = 30) group.	*L. acidophilus, L. casei, B. bifidum,* and *L. fermentum*	12 weeks	Probiotic supplementation showed favorable effects on the scores of Expanded Disability Status Scale (EDSS), improved mental health parameters, inflammatory markers, insulin resistance, HDL-, total-/HDL-cholesterol and malondialdehyde levels.	Level II	Kouchaki et al. (2017) 79
**Insomnia**	156 adults (19–65 years) with subclinical symptoms of depression, anxiety, and insomnia were randomly assigned to receive either supplement ( *n* = 78) or a placebo ( *n* = 78).	*L. reuteri* NK33 and *B. adolescentis* NK98	8 weeks	Probiotic supplementation improved sleep quality, especially sleep induction.	Level II	Lee et al. (2021) 80
**Depression**	40 participants (between 20 and 40 years) with self-reported insomnia were randomly assigned into 2 groups, probiotic, or placebo group.	*Lactobacillus plantarum* **PS128**	30 days	Daily probiotic supplementation may improve depressive symptoms and sleep quality of insomniacs.	Level II	Ho et al. (2021) 81
	110 patients with major depressive disorder (MDD) (aged 36.5 ± 8.03). Subjects were randomly assigned to receive probiotic ( *n* = 38), prebiotic ( *n* = 36), or placebo ( *n* = 36).	*L. helveticus* and *B. longum.* **Prebiotic treatment:** *Galactooligosaccharide*	8 weeks	The subjects with MDD that received probiotic supplements showed an improvement in Beck Depression Inventory (BDI) score compared with placebo, while no significant effect of prebiotic supplementation was observed.	Level II	Kazemi et al. (2019) 82
	105 obese participants (18–55 years old), were included in a double-blind, randomized, placebo-controlled trial that included a 12-week weight loss period based on moderate energy restriction, followed by 12 weeks of weight maintenance. During the two phases, subjects received probiotic formulation ( *n* = 62) or placebo ( *n* = 63).	*L. rhamnosus* GMCC1.3724 **Prebiotic treatment:** Oligofructose and inulin	24 weeks	The supplementation significantly increased weight loss and decreased food craving and BDI score when comparing to placebo group, as well as a better score in the Body Esteem Scale questionnaire. In men, the supplementation improved fasting fullness and cognitive restraint.	Level II	Sanchez et al. (2017) 83
**Anxiety**	120 college students (aged between 18 and 24 years): 60 were allocated into anxiety group and 60 into control group.	*B. longum, B. lactis* , *B. adolescentis* , *Streptococcus (S.) thermophiles* , *L. acidophilus* , and *L. delbrueckii*	15 consecutive days (twice per day)	Probiotic supplementation group presented decreased anxiety scores and restored microbiota imbalance to the standard level.	Level II	Qin et al. (2021) 84
**Depression and** **anxiety**	423 postnatal women (mean age: 33 years) were allocated into HN001 ( *n* = 212, mean age 33.5 ± 4.24) or placebo ( *n* = 211, mean age: 33.7 ± 4.44) group.	*L. hamnosus* HN001	45 weeks	Postnatal women who received the probiotic had significantly lower depression and anxiety scores in the postpartum period. The offered probiotic may be convenient for the prevention or treatment of depression and anxiety postpartum symptoms.	Level II	Slykerman et al. (2017) 85
**Autism spectrum disorder (ASD)**	30 autistic children (aged 5 to 9 years), and 30 gender- and age-matched healthy controls were recruited.	*L. acidophilus, L. rhamnosus* , and *B. longum*	3 months	The probiotic supplementation significantly improved the autism severity and gastrointestinal (GI) symptoms.	Level II	Shaaban et al. (2018) 86
	85 preschoolers with ASD (mean age: 4.2 years) were randomly allocated into probiotics ( *n* = 42) or placebo ( *n* = 43).	*S. thermophilus, B. breve, B. longum, B. infantis, L. acidophilus, L. plantarum, L. para-casei, and L. delbrueckii*	6 months	The group treated with probiotics improved some GI symptoms, adaptive functioning, and sensory profiles. Results suggest positive effects of probiotics on core autism symptoms.	Level III	Santocchi E et al. (2020) 87
**Schizophrenia**	60 patients with chronic schizophrenia (mean age: 44 years) received either co-supplementation of 50,000 IU vitamin D 3 plus probiotic ( *n* = 30) or placebo ( *n* = 30).	*L. acidophilus,* *B. bifidum, L. reuteri and* *L. fermentum* plus 50,000 IU of vitamin D3	12 weeks	The probiotic significantly improved general and total Positive and Negative Syndrome Scale (PANSS) scores and, metabolic profiles.	Level II	Ghaderi et al. (2019) 88
	65 patients with schizophrenia were assigned to probiotic group ( *n* = 33, mean age: 44.8 ± 11.2) or placebo group ( *n* = 32, mean age: 48.1 ± 9.4).	*L. rhamnosus strain GG and B.animalis* subsp. *lactis* strain Bb12	14 weeks	The authors suggest that supplementation with probiotics might ameliorate GI leakage in schizophrenia patients.	Level II	Tomasik et al. (2015) 89
**Bipolar disorder (BD)**	20 euthymic individuals with BD. All participants received the probiotic supplementation for 3 months	*L. casei W56, L acidophilus W22, L. paracasei W20, B. Lactis W51 and W52, L. salivarius W24, B. bifidum W23, L. plantarum W26, Lactococcus lactis W19.*	3 months	The probiotic supplementation may improve the cognitive function, which might lead to better psychosocial, occupational, work, and financial functioning, in BD patients.	Level IV	Reininghaus et al. (2018) 90

Notes: *This level of evidence rating scheme is based on the following: Ackley BJ, Swan BA, Ladwig G, Tucker S. (2008). Evidence-based nursing care guidelines: Medical-surgical interventions. (p. 7). St. Louis, MO: Mosby Elsevier.

### Gut microbiota and sleep disorders

#### Obstructive sleep apnea

Obstructive sleep apnea (OSA) is a pathology that occurs due to a collapse of the upper airway during sleep, leading to a decrease in or interruption of ventilation. Consequently, the affected individual has hypoxia, hypercapnia (due to reduced airflow during airway obstruction), and an increase in the number of awakenings during sleep, usually associated with events, to restore breathing. 26 Obstructive sleep apnea is related to type 2 diabetes, arterial hypertension, stroke, cardiac arrhythmias, coronary diseases, and cognitive alterations. 27 28

The intermittent hypoxia that occurs in OSA can cause an increase in anaerobic organisms in the gut. This dysbiosis increases tissue permeability, inflammation, and oxidative stress, besides being linked to cardiometabolic, neurobehavioral, and GI changes often found in individuals with OSA. An increase in *Firmicutes/Bacteroidetes* and a decrease in *Actinobacteria/Proteobacteria* was observed in individuals who snore, indicating it may influence the gut microbiota. In children with OSA, there is a decrease in the diversity of the intestinal microbiota with an increase in inflammation in the intestinal barrier due to intermittent hypoxia. 29

Studies that evaluated the intestinal microbiota of individuals with OSA observed intestinal dysbiosis in these patients. 30

Experimental studies show that this dysbiosis may improve with the use of pre and probiotics. 26 28 Prebiotics and probiotics with weight loss increase the quality of life and decrease liver fat, visceral fat, abdominal circumference, interleukin 6 (IL-6), other proinflammatory cytokines, and cortisol. 28

#### Sleep deprivation

Sleep deprivation occurs when an individual sleeps less time than is necessary for rest and health maintenance. It can cause or lead to several pathologies, such as cardiovascular diseases, atherosclerosis, coronary diseases, alterations in the autonomic nervous system, immunological alterations, metabolic, respiratory, and GI disorders, cognitive, mood, and attention and memory alterations, and stroke. It is also related to increased appetite, insulin resistance, and increased obesity. 31

Sleep deprivation or sleep fragmentation are stressors capable of altering the intestinal microbiota, leading to dysbiosis, and increasing intestinal permeability, consequently increasing inflammation. 32 After acute sleep deprivation, a decrease in gut microbiota diversity was found. 28 33 In experimental models, sleep deprivation has been observed to cause oxidative stress and intestinal cell damage. 34

Microbiome diversity was correlated with sleep efficiency and wakefulness after sleep onset. We found positive correlations between total microbiome diversity and IL-6, a cytokine previously observed for its effects on sleep. 35

Studies have shown an increase in the abundance of *Firmicutes* related to sleep efficiency and a reduction in the quantity of *Bacteroidetes* in the intestinal microbiota in sleep-deprived individuals. This change is associated with obese individuals and could explain why sleep-deprived individuals tend to gain weight. 35

Another sleep disorder that is associated with decreased sleep time is insomnia. Reduced bacterial diversity could be related to acute and chronic insomnia. Patients with the condition showed a decrease in anaerobic microorganisms. There is an alteration in the microbiome structure of patients with chronic insomnia, and there may be a decrease in butyrate-producing microorganisms and a consequent increase in inflammatory markers. There was also an increase in the genus *Blautia* and a reduction in *Faecalibacterium* , which could be associated with inflammatory changes and neuropsychological diseases, including diabetes and GI disorders. 36 It is also observed that sleep-deprived patients may have decreased colon and stool melatonin levels. 37

#### Circadian rhythm and gut microbiota

The human body follows the circadian rhythm for around 24 hours. The sleep-wake period's circadian rhythm follows endogenous patterns independent of external and exogenous factors such as daylight. The interruption in the circadian rhythm leads to a proinflammatory state and increases the risk of cardiovascular diseases, diabetes, and metabolic syndrome, among others. 38

The circadian rhythm influences the intestinal bacteria population, fluctuating during the day. 39 The feeding schedule is an important marker off- and impact on- the rhythm, and when the former changes, so does the latter. With the interruption of the circadian rhythm, dysbiosis occurs in the intestinal microbiota, and researchers could detect changes in cell permeability and inflammatory and immune responses. 40

It was observed that, in experimental models, the interruption of the circadian rhythm of animals alters functions, such as the rhythmicity of the intestinal microbiota, and causes changes in their genetics. Thus, there is a change in the composition of this microbiota. With dysbiosis, there is a change in intestinal permeability and increased inflammation, with possible harmful health effects. The observed effects are increased glucose intolerance and weight gain. 34

#### Microbiota, other sleep disorders, and related comorbidities

Other sleep disorders that worsen sleep quality could be related to changes in the intestinal microbiota. Even neurological diseases and cognitive alterations are related to sleep and could be related to intestinal alterations. A study showed that changes in sleep and consequently in the microbiota could be a factor that would bring cognitive changes to the elderly. 41

The intestinal microbiota of children with autism spectrum disorder and with sleep problems was unbalanced, with an increase in *Faecalibacterium* and a decrease in *Agathobacter* . There was a decrease in the production of butyrate and its metabolites, worsening sleep and aggravating other central symptoms in these individuals. 42

No differences were found in patients with type 1 narcolepsy relative to controls, between groups for microbial diversity, population richness, and uniformity. 43

In studies with bipolar patients, a relationship was found between *Faecalibacterium* and *Lactobacterium* and sleep quality, suggesting that increasing these two types could improve sleep in these patients ( Table 1 ). 44

### Psychiatric disorders

#### Depression

Before the COVID-19 pandemic, the global prevalence of depression and anxiety disorders was alarming. The World Health Organization (WHO) estimated that, globally, the number of individuals with depression in 2015 exceeded 300 million. 45 Nevertheless, research performed in specific countries showed that the incidence of anxiety and depression has increased significantly throughout the COVID-19 pandemic. According to the scientific brief released by the WHO, the global prevalence of anxiety and depression increased by 25%. 46

Although antidepressants, the primary drugs used for depression, are often adequate to treat this disabling mental disorder, it often causes side effects. 47 Since side effects increase with longer-term use, and antidepressants, in most cases, should not be taken for more than 2 years, patients and their physicians usually desire to get off the medication as the depression symptoms get better. 48

There is increasing use of pre and probiotics with psychotherapy, such as complementary and alternative therapies for mental disorders. 49 It is well known that the microbiota-gut-brain axis has a bidirectional communication and microbiota imbalance leads to adverse effects on central nervous system functions that increase the symptoms and mental illness. 50 Although the mechanism by which the gut microbiota plays a role in mental health is still controversial, researchers suggest that factors such as the hypothalamic-pituitary-adrenal (HPA) axis and serotonergic, GABAergic, and other neurotransmitters signaling systems are probably the central part of this correlation. 51

The HPA axis is the human central stress response system, and hyperactivity is identified in several patients diagnosed with depression and is activated by gut dysbiosis through specific mechanisms. 52

Gut dysbiosis can also be related to depression symptoms, impacting tryptophan metabolism and the serotoninergic system. Tryptophan (an essential amino acid) is the exclusive precursor of serotonin, and the human intestinal microbiota has a crucial role in regulating the tryptophan metabolism. 53 Additionally, the GI tract has high levels of 5-hydroxytryptamine (5-HT), and studies suggest that the epithelial enterochromaffin (ECCs) cells are responsible for 90% of 5-HT secretion, while only 10% remains from the enteric nervous system. 54 Bacteria, such as *Escherichia, Streptococcus, Enterococcus,* and *Candida* , are some gut microorganisms capable of producing serotonin. 55 Brain functions can be affected by gut dysbiosis, as intestinal microorganisms influence not only serotonin synthesis/metabolism but also the synthesis of other neurotransmitters. 4

#### Anxiety

Anxiety symptoms are frequent in patients with GI imbalance or GI disorders. For instance, more than 50% of patients diagnosed with irritable bowel syndrome have anxiety or depression as a comorbidity. 56

The correlation between anxiety symptoms and the alteration of gut microbiota diversity and complexity has been extensively documented. The factors implicated in this correlation are due to the role of gut microbiota and brain mutual signals, including neurotransmitter systems and immunologic factors. The mechanisms responsible for the close relationship between gut dysbiosis and anxiety are similar to those that correlate with gut microbiota imbalance and depression. Hypothalamic-pituitary-adrenal axis activation is the protagonist in stress and anxiety symptoms by activating and releasing adrenocorticotropic hormone (ACTH), which stimulates the production of glucocorticoids (e.g., cortisol) into the bloodstream. 57 The intestinal microbiota imbalance leads to activation of the HPA axis, which provokes mental condition alterations and mental disorders such as anxiety. 58

Although the anxiety pathophysiology is not completely clear, it is well known that neurotransmitters, including serotonin, dopamine, and GABA, are linked to anxiety disorders, and intestinal microbiota has been shown to alter all these neurotransmitter modulations in the brain. There is growing evidence indicating that this impairment triggered by dysbiosis can also explain the relationship of gut microbiota with anxiety disorder. 59

Besides tryptophan and serotonin modulation, several experimental studies have demonstrated that gut microbiota similarly plays a role in dopamine modulation, one of the most important neurotransmitters involved in anxiety symptoms in distinct brain parts. 60

#### Autism spectrum disorder (ASD)

Numerous studies have already demonstrated that patients with ASD suffer from several gut-related comorbidities, including diarrhea, constipation, nausea, abdominal pain, vomiting, and reflux. Besides, their microbiota is distinctive from healthy subjects, and gut dysbiosis is much more frequent. 61

Studies have been showing that the chances of developing ASD are higher in some situations that trigger dysbiosis in early childhood, including children born by cesarean, children who were not breastfed, those who were treated with antibiotics during early childhood, children born to a mother that used antibiotics during pregnancy, and those whose mother had to be hospitalized due to infection when pregnant.

In a recent meta-analysis, researchers observed that reduced quantities of *Streptococcus* and *Bifidobacterium* were associated with ASD diagnosis. In contrast, another meta-analysis found that people with ASD present an intestinal microbiota with increased concentration of *Bacteroides* and *Clostridium* and decreased amounts of *Bacteroidetes, Firmicutes* , and *Proteobacteria* phyla. 62

#### Schizophrenia and bipolar disorder

Similar to what has been observed in ASD, studies suggest that imbalance that might happen in newborns' gut microbiota seems to be associated with an increased risk of developing schizophrenia. Moreover, these patients also usually suffer from GI disorders. 63

Studies have shown that the infection of the gut microbiota by the protozoa *Toxoplasma gondii* seems to cause main changes in the intestinal composition, which are associated with an environmental risk factor for the onset of schizophrenia and bipolar disorder (BD). 64 The differences in gut microbiota diversity and quantity of patients with schizophrenia are still unclear as some studies show no difference, while others observed distinct variations. Elevated levels of *Lactobacilli* appear to be the only alteration that is consistent among studies. 63

Concerning the intestinal microbiota composition of patients with BD, it is also unclear how discrepant it is compared with healthy volunteers. 65 Nevertheless, studies show that it seems to be altered, as a lower amount of *Faecalibacterium* and *Ruminococcaceae* and higher levels of *Actinobacteria* and *Coriobacteria* were found when compared with healthy controls. 66 Another observation that implies an association between BD and dysbiosis is that evidence shows that hospitalized patients with acute mania received antimicrobial medications nearly twice as much as control patients, as they have an elevated rate of bacterial infections ( Table 1 ). 67

### Dysbiosis treatment

The first guidance health professionals must understand and disseminate is the importance of maintaining a delicate gut microbiota balance as the top priority goal of dysbiosis prevention. Thus, factors related to microbiota imbalance that can consequently affect or even lead to the development of neuropathologies should be part of physicians' knowledge and orientation. 68

The factors that can negatively impact gut microbiota include delivery methods (cesarean sections); short, or absence of, breastfeeding (inclination for formula-feeding); early introduction of solid food (3 months of age or earlier); antibiotic use during pregnancy; antibiotics use in early life; and poor dietary habits and food choice (poor diets), including high consumption of ultraprocessed food, high rates of antibiotics consumption during life, drug use, smoking tobacco or electronic cigarettes, alcohol consumption, and sedentarism. 68

It is essential to highlight that early infancy is a relevant intestinal microbiota colonization period. Thus, raising awareness about gut microbiota during pregnancy and in the earliest years of life (at least until 3 years of age) is necessary to avoid dysbiosis, which is lined to extraintestinal diseases, such as neurological ones. 68

Another strategy to treat gut dysbiosis includes dietary modification. 69 Dietary methods to treat dysbiosis and keep a healthy gut microbiota composition include limiting processed food as much as possible (Mediterranean diet). Furthermore, diets should incorporate fermented food that is rich in probiotics (e.g., kefir, yogurt with live active cultures, pickled vegetables, fermented milk products, kombucha tea, kimchi, and miso), high-fiber foods (e.g., fresh fruits, whole grains, vegetables, legumes, and beans), spices, and foods that are rich in polyphenols, oleic acids, and omega-3 fatty acids. Patients should avoid the consumption of saturated fatty acids, refined carbohydrates, and high protein and/or sugar intake.

Polyunsaturated fatty acids (PUFAs), especially omega 3, are essential in the human diet. A few studies have described the benefit of omega 3 in patients with PD. Although it is a good and safe profile with ease of administration, more studies with PUFAs, controlling for all possible covariates, must be done. 70

Antibiotics can inhibit or eliminate some microorganisms, causing the vanishing of some and the advent and progression of new bacterial species, with consequent dysbiosis. The knowledge about the relationship between the coexisting species and how they interact with their host is a challenge for new therapeutic approaches to treat dysbiosis. 71 The action of antibiotics, such as ceftriaxone, moxycycline, minocycline, and rifampicin, has been related to gut dysbiosis and neuroprotection. Still, the long-term use due to toxicity and resistant bacterial selection is a risk in clinical practice. 72

The probiotic (active microorganisms with health benefits), prebiotic (non-digestible food ingredients that stimulate the activity of particular microorganisms), and symbiotic (combination of probiotics and prebiotics) supplementation is a potential therapeutic intervention for gut dysbiosis, and it is considered safe for most of the population ( Table 1 ). 69 73 74 75 76 77 78 79 80 81 82 83 84 85 86 87 88 89 90

As shown in Table 1 , most of the revised manuscripts were randomized-controlled trials that administrated probiotics in patients diagnosed with brain, psychiatric, or sleep disorders, and most of them showed a high level of evidence (level II) of probiotics to treat gut dysbiosis and alleviate the symptoms of the studied disease, with no side effects or toxicity. Although the results are promising, the therapeutic approaches need to be better understood in relation to the mechanisms involved.

Psychobiotics are a type of probiotics that, when consumed in appropriate quantity, have the potential to improve mental health. Psychobiotics may improve mental health by downregulating the HPA axis, decreasing proinflammatory cytokines levels, attenuating the disruption of the BBB, enhancing the production of neurotransmitters such as serotonin and GABA, and modulating neurotransmitter balancing. These same types of probiotics might be beneficial for patients with sleep disorders, serving to alleviate symptoms by the same mechanisms. 91

Live biotherapeutic products (LBPs) are distinguished from probiotic supplements because they have evidence for treating or preventing diseases. They can be the result of engineering by addition, erasing, or adjusting genetic material within the organisms ( *E. coli* , *Bacteroides* , and lactic acid bacteria). 92 Synthetic biology has enormous potential to contribute to treating patients with neurological diseases. 93

Fecal microbiota transplantation (FMT) is a new promising therapeutic option for gut dysbiosis. Numerous studies have shown that transplanting feces from a healthy control into a patient diagnosed with a neurological disorder can alleviate the pathological conditions, signs, and symptoms. 94 95 In contrast, experimental studies showed that when researchers transferred fecal bacteria from a donor with a neurological disorder to a healthy recipient, the method induced the appearance of similar symptoms in the healthy host. 51 94 Nevertheless, further studies are still necessary to validate this novel therapeutic method in humans with neurological disorders, as FMT can revolutionize the treatment of conditions other than GI diseases.

In conclusion, the increasing evidence about the existence of a close and bi-directional correlation between the gut microbiota and the brain indicates that dysbiosis plays an essential role in the development, function, and disorders of the CNS. Although more studies are still necessary, our review demonstrates that avoiding dysbiosis, especially during pregnancy and early childhood, is essential to prevent neurological disorders. Moreover, using probiotics by patients with psychiatric or brain diseases is safe in most cases. It can treat gut dysbiosis, decrease depression and anxiety scores, and alleviate the symptoms and severity of the studied neurological disorders. These observations point out the potential and safety of probiotics and prebiotics as adjuvants in preventing and treating neurological diseases.
